# Reflection and observation: cell-based screening failing to detect HBV in HUMSCs derived from HBV-infected mothers underscores the importance of more stringent donor eligibility to reduce risk of transmission of infectious diseases for stem cell-based medical products

**DOI:** 10.1186/s13287-018-0920-3

**Published:** 2018-07-04

**Authors:** Wei Liu, Yuanyuan Xie, Tianyun Gao, Feifei Huang, Liudi Wang, Lijun Ding, Wenqing Wang, Shuo Liu, Jianwu Dai, Bin Wang

**Affiliations:** 1 Clinical Stem Cell Center, The Affiliated Drum Tower Hospital of Nanjing University Medical School, 321 Zhongshan Road, Nanjing, 210008 China; 2 Institute of Genetics and Developmental Biology, Chinese Academy of Sciences, No. 1 West Beichen Road, Beijing, 100190 China

**Keywords:** Human umbilical cord mesenchymal stem cells, Cell-based therapy, Clinical screening assay, Hepatitis B virus, Serological test, Droplet digital PCR

## Abstract

**Background:**

In cell-based therapy, the transmission of communicable diseases imposes a substantial threat to recipients. In this study, we investigated whether cell-based screening could detect hepatitis B virus (HBV) in human umbilical cord-derived mesenchymal stem cells (HUMSCs) isolated from HBV-infected donors to understand the susceptibility of HUMSCs to HBV infection.

**Methods:**

HBV assay was performed in HUMSCs derived from healthy and HBV-infected donors with enzyme-linked immunosorbent assay (ELISA), fluorescence quantitative PCR (FQ-PCR) assay, and droplet digital PCR (ddPCR) assay. Further, HBV DNA was assayed in HUMSCs derived from healthy donors after incubation with human sera containing a high titer of HBV using FQ-PCR.

**Results:**

HBV antigen/antibody and DNA failed to be detected using ELISA, FQ-PCR, and ddPCR. After incubation with HBV infection sera, HBV DNA could be detected, but below the valid titer of the assay kit. The HBV DNA levels in HBV-incubated HUMSCs gradually decreased with medium change every 2 days and then significantly decreased, not even detected after passage.

**Conclusions:**

The current cell-based screening methods could not detect HBV in HUMSCs derived from HBV-infected donors, indicating the importance of more stringent donor eligibility to reduce the risk of transmission of communicable diseases in cell-based therapy. To solve the problem of an occult HBV window period in donor eligibility determination, we recommend that the donors undergo another HBV serological test 3 months after the first serological communicable disease screening.

## Background

In recent decades, mesenchymal stem cell (MSC)-based therapy of various diseases is attracting more and more attention. Multiple organs and tissues have been reported to be the sources of MSCs such as umbilical cord (UC) [1], bone marrow (BM) [2], adipose tissue (AD) [3], amniotic membrane [4], dental pulp [5], placenta [6], and even urine [7]. Human umbilical cord-derived mesenchymal stem cells (HUMSCs) have the advantages of easier preparation, lower immunogenicity [8, 9], higher proliferation [10], and multilineage differentiation capability [11]. Therefore, HUMSCs have been widely used in clinical trials for acute-on-chronic liver failure [12], systemic lupus erythematosus [13], children’s leukemia [14], graft versus host disease (GVHD) [15], and so on. To date, MSCs have been used in over 220 clinical trials worldwide (www.clinicaltrials.gov), 10% of which were HUMSCs [16]. With the continuous increase of clinical application of MSCs, researchers are faced with the challenge of ensuring safety and effectiveness of stem cells for human use.

Based on the existing stem cell guidelines [17] and established regulatory systems regarding cell therapeutic products in the USA and China [18–20], pathogen contamination must be excluded such as potential bacterial, fungal, mycoplasma, and viral contamination in transplanted stem cells. Thus, infectious pathogens like hepatitis B virus (HBV), hepatitis C virus (HCV), human immunodeficiency virus (HIV), syphilis, and cytomegalovirus (CMV) must be screened for in peripheral blood from donors for donor eligibility determination and in derived stem cells for safety [21]. HBV, one of the most common infectious diseases, especially in China, infects more than 2 billion people worldwide. Hepatitis B is a leading cause of chronic hepatitis, cirrhosis, and hepatocellular carcinoma, accounting for 0.6 million deaths annually. HBV represents a substantial threat to public health. Sexual or household contact or unsafe injection is a vertical route of HBV transmission. HBV can also transmit from a chronically infected woman to her infant during production [22]. With the advances in molecular techniques, the presence of hepatitis B surface antigen (HBsAg) and HBV DNA has been described in peripheral blood mononuclear cells (PBMCs) [23], lymphoblastoid cells [24], lymph nodes [25], and fluids such as spermatozoa, urine, and saliva [26] in patients with HBV infection. In addition, accumulating evidence reported the transmission of HBV caused by tissue transplantation [27–32] and by hematopoietic stem cells and blood with hepatitis B surface antibody (anti-HBs)-positive/HBsAg-negative blood [33–36]. By analogy, there is a potentially similar HBV transmission risk for patients receiving HUMSC transplantation if the HUMSCs were derived from HBV donors.

Thus, the specific regulation of donor eligibility determination has been built up to reduce the risk of transmission of infectious diseases for stem cell-based medical products by national authorities. According to current regulations and clinical practices, both MSCs from hepatitis B carriers and final cell products positive for HBVs must be discarded. The rationale for secondary HBV testing of the derived cells after the blood test screening is that derived HUMSCs are potentially positive for HBV DNA during the window period in HBsAg-negative blood specimens. Namely, if the donor eligibility is determined during the HBV window period by serological screening tests, the donor will be considered negative for HBV infection and a qualified donor. But, in fact, the final HUMSCs are derived from an HBV carrier. Thus, after blood test screening, adding an HBV test on the derived cells is considered to provide a potential reduction of the residual risk of transmission of HBV infection in clinical settings. However, whether HUMSCs from HBV carriers are hosted by HBVs and whether the current clinical cell-based routine screening could successfully provide a potential reduction of the residual risk of transmission of HBV infection in clinical settings remain elusive.

In our study, enzyme-linked immunosorbent assay (ELISA), fluorescence quantitative PCR (FQ-PCR) assay, and droplet digital PCR assay (ddPCR) were used to detect HBV in HUMSCs and their culture medium derived from patients with or without HBV infection. Our objective was to find the susceptibility of HUMSCs to HBV infection and whether it is applicable to detect HBV using existing detection assays in HUMSCs derived from donors with HBV infection. We analyzed the current screening strategy to find the critical points to reduce HBV transmission risk from stem cell-based therapy and optimized the process to reduce the occurrence of stem cell-associated infectious diseases so that stem cells can have more applications.

## Methods

### Isolation and culture of human HUMSCs

This study was approved by the Research Ethics Board of Nanjing Drum Tower Hospital. Written consent was obtained from the parents. Before delivery, maternal blood was harvested for HBV serological screening examination. After full-term births, fresh UC was aseptically stored in sterile PBS and processed within 4 h after partum. HUMSCs were isolated from UC using the tissue explant method [37]. Briefly, the major blood vessels in fresh UC were removed in a laminar flow clean cabinet. After washing three times with 1 × PBS, the remaining connective tissue (Wharton’s jelly) was cut into small pieces of approximately 1 mm^3^. The tissue pieces were placed in a T75 cell culture flask (Corning, NY, USA) and kept in a 5% CO_2_ incubator at 37 °C without medium for 4 h. Then, 5 ml DMEM medium with 10% fetal bovine serum (FBS) was gently added to the culture flask. The culture medium was changed every 3–4 days. When well-developed colonies of fibroblast-like cells appeared, the cells were trypsinized and transferred into a new flask for further expansion. The cells were passaged three times to remove the contamination of other type cells and MSCs at the third passage were used for all of the experiments.

### Flow cytometry analysis

For phenotypic analysis, 100,000 cells at the third passage were incubated with human monoclonal antibodies labeled with either fluroisothiocyanate (FITC) or phycoerythrin (PE) (CD11b FITC, CD19 PE, CD34 FITC, CD44 PE, CD45 FITC, CD73 PE, CD90 FITC, CD105 PE, HLA-DQ FITC, and HLA-DR PE; BD, San Diego, CA, USA) in the dark for 30 min at room temperature. After washing three times with 1 × PBS, HUMSCs were centrifuged at 2000 rpm for 5 min and resuspended in 1 × PBS for flow cytometry analysis. Cells were analyzed using a FACScan (BD FACSAria™; BD,San Jose, CA, USA) and data were analyzed with FACS software.

### Adipogenic and osteogenic differentiation

HUMSCs were cultured in a 24-well tissue culture plate at a density of 1 × 10^4^ cells/well using DMEM with 10% FBS. When the cells reached 50–70% confluency, the medium was replaced with adipogenic (Gibco, Grand Island, NY, USA) and osteogenic (Gibco, Grand Island, NY, USA) differentiation medium to induce adipogenesis and osteogenesis, respectively. The differentiation medium was changed every 3–4 days. After 21 days, adipogenesis and osteogenesis differentiation were assayed using Oil red O staining (Sigma-Aldrich, St. Louis, MO, USA) and Alizarin red-S staining (Sigma-Aldrich, St. Louis, MO, USA), respectively.

### Serological detection of HBV

The serological examination of HBV in maternal blood was performed using an ELISA kit (WANTAI Biopharm, Beijing, China) with the automatic enzyme immunoassay analyzer (FAME 24/20; Hamilton, Switzerland)**.**

### Detection of hepatitis B surface antigen and hepatitis B e-antigen in HUMSCs

The media from the primary and third-passage cultures of HUMSCs were collected and stored at − 80 °C for HBsAg and hepatitis B e-antigen (HBeAg) detection. HUMSCs at the third passage were trypsinized and washed three times with 1 × PBS to ensure the removal of culture medium. Then, HUMSCs were resuspended in 300 μl of 1 × PBS. The HUMSCs were disrupted with ultrasonic cell crushers (VCX-130; SONICS, USA) and the supernatant was collected. The HBsAg and HBeAg amounts in medium and cell crushing supernatant were assayed using an ELISA kit with the automatic enzyme immunoassay analyzer.

### HBV DNA assay

To further assay intracellular and extracellular HBV DNA, the culture medium in HUMSCs at the primary and third passages as well as aliquots of the cells (10^7^ cells) at the third passage were collected. The total DNA in cells and culture medium were extracted according to the protocol of the TIANAMP Genomic DNA Kit (TIANGEN, Beijing, China). Quantitative PCR was performed to detect HBV DNA using a kit (DAANGENE, Canton, China). To determine the testing limit of HBV DNA, the standard HBV template (a viral load of 10^8^ IU/ml provided by the kit) was diluted to 10^0^–10^8^ IU/ml and tested using FQ-PCR. As Fig. 2 shows, HBV DNA ≥ 100 IU/ml was considered a valid assay result.

### Droplet digital PCR assay

ddPCR, the next-generation technology of quantitative PCR methods, offers high precision and absolute quantification of target nucleic acid without reference standard curves, and can reach a detection limit lower than FQ-PCR [38]. The quantification of HBV copy number in culture medium and cell lysate of HUMSCS at the third passage was assayed using the QX200™ Droplet Digital™ PCR system (1864001; Bio-Rad, USA). Briefly, ddPCR reaction solution was prepared as follows. The 20 μl reaction mixture comprised 2 × ddPCR™ Supermix (1863023; Bio-Rad, USA), 1.8 μl HBV sense (5′-TGGTGTCTTTTGGAGTGTGGAT-3′) and antisense (5′-TAACATTGAGATTCCCGAGATTG-3′) primers, 0.5 μl HBV probe (5′-FAM-TCTTCTGCGACGCGGCGATT-TAMRA-3′), 2 μl of DNA sample, and 3.9 μl RNase/DNase-free water. The mixture was transferred to the DG8 cartridge and then 70 μl of droplet generation oil (1863005; Bio-Rad, USA) was added, and droplets were generated by the QX200™ Droplet Generator (1864002; Bio-Rad, USA). The droplets were transferred to the 96-well PCR response plate and amplified on the C1000 Touch™ thermal cycler (1851197; Bio-Rad, USA). The cycling conditions were 95 °C for 10 min, followed by 40 cycles of 94 °C for 30 s and 55 °C for 60 s, final incubation for 10 min at 98 °C, and ending at 4 °C. After amplification, the 96-well plate was placed into the Droplet Reader (1864003; Bio-Rad, USA) and the data were analyzed automatically by the QuantaSoft analysis software (Bio-Rad, USA), the results presented as copy number per microliter. In order to verify the sensitivity and accuracy of the ddPCR amplification system, we repeated the experiment using a commercialized primer probe (Fosun Pharma, Shanghai, China), and the HBV DNA of known concentrations extracted from patient serum samples was diluted to serial concentrations for detecting the limit of ddPCR. RNase/DNase-free water was used as a negative control.

### HBV detection in HUMSCs incubated with HBV-positive serum

To investigate whether HBV is capable of hosting MSCs, we incubated MSCs with human sera containing HBV and then detected HBV in MSCs. The HUMSCs derived from a healthy donor (No. 3) were seeded in a six-well culture plate at a density of 1 × 10^5^ cells/well. After 24 h, the culture medium was changed with DMEM-Lg + 10% human blood sera derived from an HBV infectious patient who was positive for HBsAg, HBeAg, and HBcAb, as well as a serum HBV DNA load of 9.34 × 10^7^ IU/ml. Following incubation for 24 h, culture medium was discarded and HUMSCs were washed with 1 × PBS five times. At the end of the last wash, washing PBS and a part of the HUMSCs (day 0) were collected for detection of HBV DNA using FQ-PCR. The remaining HUMSCs were cultured with complete medium and were divided into three groups (no medium change and no passage, medium change every 2 days, and passage every 2 days). Aliquots of HUMSCs (10^6^ cells) and culture supernatants were collected at different time points for detecting HBV DNA using FQ-PCR. HUMSCs incubated with human sera from healthy donor were used as negative controls.

### Statistical analysis

The measurement data were expressed by mean ± standard deviation (SD). The statistical analysis of data was performed using GraphPad prism 6 software (GraphPad Software, USA). The difference between data derived from the different groups was performed by multiway analysis of variance (ANOVA). *p* < 0.05 was considered statistically significant.

## Results

### Serological HBV screening in 14 maternal blood samples

In the present study, 14 maternal blood samples from donors were collected before delivery and were investigated for serological HBV markers including HBsAg, anti-HBs, anti-HBsAg, HBeAg, anti-HBe, and anti-HBc using ELISA (Table 1). Serological examinations showed that two donors (No. 1 and No. 2) were positive for HBV infection and the others were healthy (No. 3–No. 14). To investigate whether HBV could be detected in HUMSCs derived from HBV-infected women, we isolated and cultured HUMSCs derived from 12 healthy and two HBV-infected women.

**Table 1:** Serological HBV markers for 14 maternal blood samples

Sample No.	HBsAg	Anti-HBs	HBeAg	Anti-HBeAg	Anti-HBc
1	+	–	–	+	+
2	+	–	+	–	+
3	–	+	–	–	–
4	–	–	–	–	–
5	–	+	–	–	–
6	–	–	–	–	–
7	–	–	–	–	–
8	–	+	–	–	–
9	–	+	–	–	–
10	–	+	–	–	–
11	–	+	–	–	–
12	–	+	–	–	–
13	–	+	–	–	–
14	–	+	–	–	–

*HBV* hepatitis B virus, *HBsAg* hepatitis B surface antigen, *Anti-HBs* antibody to HBsAg, *HBeAg* hepatitis B e-antigen, *Anti-HBe* antibody to HBeAg, *Anti-HBc* antibody to hepatitis B core antigen

### Characterization of HUMSCs

**Fig. 1:**
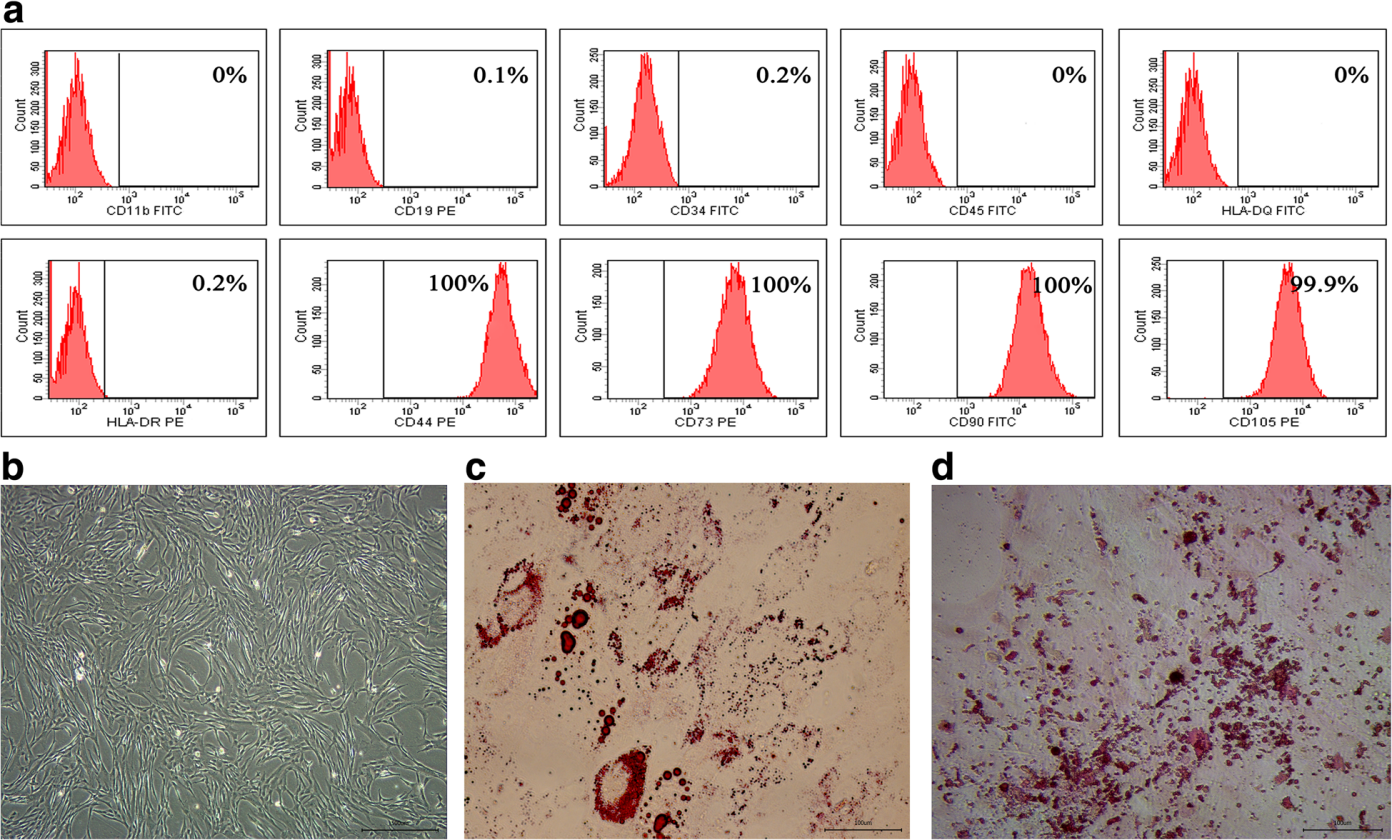
Characterization of HUMSCs. **a** HUMSCs derived from a healthy donor (No. 3) positively expressed CD44, CD73, CD90, and CD105, but negatively expressed CD11b, CD19, CD34, CD45, HLA-DQ, and HLA-DR by flow cytometry analysis. **b** Morphology of HUMSCs under light microscope. Scale bars = 500 μm. **c**, **d** Oil red O staining and Alizarin red-S staining showed HUMSCs were induced into adipogenic and osteogenic cells, respectively. Scale bars = 100 μm

HUMSCs derived from healthy donors displayed a homogeneous fibroblast-like morphology (Fig. 1b). HUMSCs positively expressed markers of CD44, CD73, CD90, and CD105, and negatively expressed CD11b, CD19, CD34, CD45, HLA-DQ, and HLA-DR surface markers (Fig. 1a). HUMSCs had a powerful committed differentiation potential of adipogenic and osteogenic lineages (Fig. 1c, d). HUMSCs derived from HBV-infected donors had similar positive surface markers and committed differentiation capability (data not shown). These characterizations showed that the isolated cells were in line with the minimum standards of stem cell proposed by the International Society for Cellular Therapy and excluded the contamination of other cells.

### Failed detection of HBV in HUMSCs derived from HBV donors

**Fig. 2:**
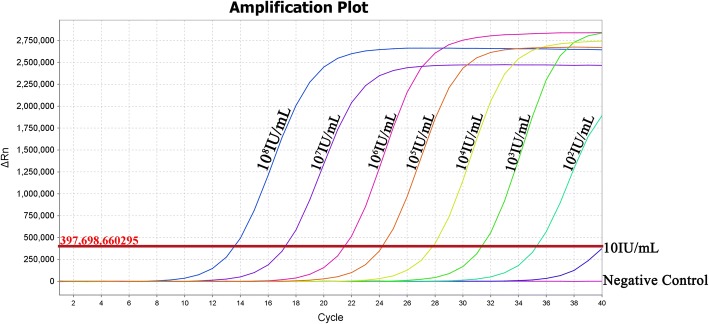
Amplification curve of HBV diluted standard. The value of ΔRn indicates the amount of probe degradation during PCR, which is the amount of PCR product. Valid detection limit of HBV PCR fluorescence quantitative detection kit was 100 IU/ml

We collected the media in the primary and third-passage cultures of HUMSCs derived from healthy (No. 3) and HBV-infected donors (No. 1 and No. 2) for screening HBsAg, anti-HBs, HBeAg, anti-HBe, and anti-HBc using ELISA and for detecting HBV DNA using FQ-PCR, as well the lysate supernatant of HUMSCs at the third passage (Table 2). As expected, we did not detect HBsAg, anti-HBs, HBeAg, anti-HBe, anti-HBc, and HBV DNA in the medium and cell lysate of HUMSCs derived from the healthy donor (No. 3). However, we also failed to detect HBV in the medium and cell lysate of HUMSCs derived from HBV-infected donors (No. 1 and No. 2). The standard curve of HBV diluted standard is shown in Fig. 2, the detection limit of the HBV PCR fluorescence quantitative detection kit is 100 IU/ml. Individual samples with HBsAg positivity or HBV DNA ≥ 100 IU/ml were considered positive for HBV infection according to the kit instructions.

**Table 2:** Failed detection of HBV in culture medium and lysate supernatant of MSCs derived from HBV-infected donors

Donor		HBsAg	Anti-HBs	HBeAg	Anti-HBeAg	Anti-HBc	HBV DNA
No. 1	1st passage medium	–	–	–	–	–	–
	3rd passage medium	–	–	–	–	–	–
	3rd passage cell lysate	–	–	–	–	–	–
No. 2	1st passage medium	–	–	–	–	–	–
	3rd passage medium	–	–	–	–	–	–
	3rd passage cell lysate	–	–	–	–	–	–
No. 3	1st passage medium	–	–	–	–	–	–
	3rd passage medium	–	–	–	–	–	–
	3rd passage cell lysate	–	–	–	–	–	–

*HBV* hepatitis B virus, *MSC* mesenchymal stem cell, *HBsAg* hepatitis B surface antigen, *Anti-HBs* antibody to HBsAg, *HBeAg* hepatitis B e-antigen, *Anti-HBe* antibody to HBeAg, *Anti-HBc* antibody to hepatitis B core antigen

### Droplet digital PCR assay

As shown in Table 3, the minimum detection limit of the ddPCR system for detecting HBV DNA template was as low as one copy of DNA. The medium and the cell lysate at the third passage derived from a healthy donor (No. 3) and HBV-infected donors (No. 1 and No. 2) were collected for assaying the HBV copy number using ddPCR (Table 4). We did not detect HBV DNA in the medium and cell lysate of HUMSCs derived from the healthy donor (No. 3). We also failed to detect the HBV copy number in the medium and cell lysate of HUMSCs derived from HBV donors (No. 1 and No. 2).

**Table 3:** Detection limit of ddPCR for HBV DNA

Dilution	Concentration (copies/μl)	
	Self-designed primer probe	Commercial primer probe
1×	3710	3246
10×	402	343
10^2^×	42.8	32.9
10^3^×	4.1	3.9
10^4^×	0.58	0.21
10^5^×	0.06	0
10^6^×	0	0.06
10^7^×	0	0

*ddPCR* droplet digital PCR, *HBV* hepatitis B virus

**Table 4:** Failed detection of HBV DNA in culture medium and cell lysate of MSCs derived from HBV-infected donors using ddPCR

Donor		Concentration (copies/μl)	
		Self-designed primer probe	Commercial primer probe
No. 1	3rd passage medium	0	0
	3rd passage cell lysate	0	0
No. 2	3rd passage medium	0	0
	3rd passage cell lysate	0	0
No. 3	3rd passage medium	0	0
	3rd passage cell lysate	0	0

*HBV* hepatitis B virus, *MSC* mesenchymal stem cell, *ddPCR* droplet digital PCR

### HBV detection in HUMSCs incubated with HBV-positive serum

**Fig. 3:**
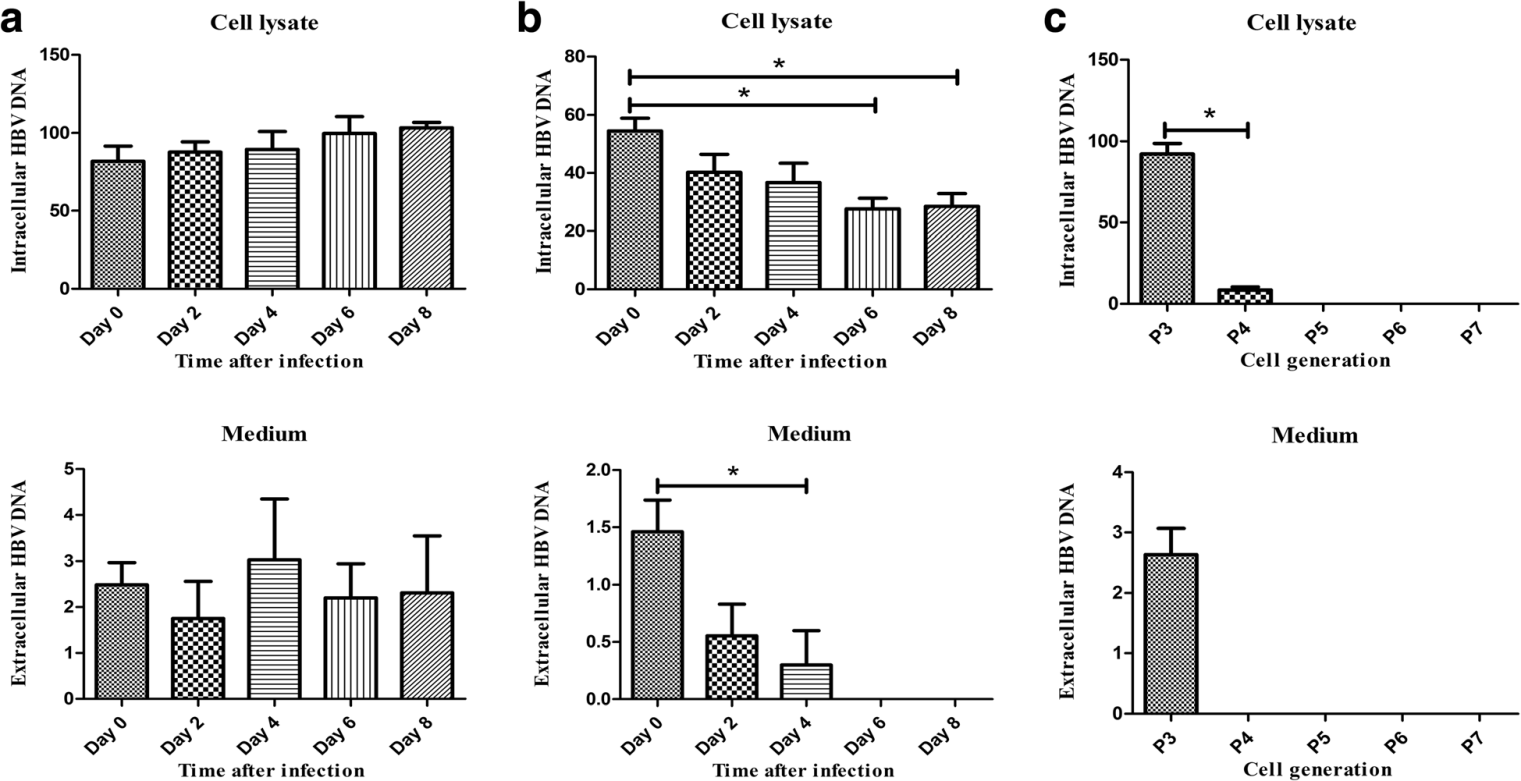
HBV DNA detection in HUMSCs after incubation with human sera with HBV using FQ-PCR. **a** HBV DNA detection in HUMSCs without medium change and passage after incubation with human sera from HBV-infected donor. **b** HBV DNA detection in HUMSCs with medium change every 2 days. HBV DNA levels gradually decreased in cell lysate and medium from day 0 to 8. **c** HBV DNA detection in HUMSCs with passage. In cell lysate, HBV DNA level in P3 HUMSCs significantly higher that than in P4 HUMSCs, and failed to be detected in P5–P7 HUMSCs. In culture medium, HBV DNA detected only in P3. HBV hepatitis B virus, * represents *p* < 0.05 and *p* < 0.05 was considered statistically significant

After HUMSCs were incubated with human sera with HBV, HBV DNA could be detected in medium and cell lysate of HUMSCs at indicated time points without medium change and passage treatment (Fig. 3a). The levels of HBV DNA in cell lysate clearly were higher than that in the medium. For HUMSCs with medium change every 2 days, the HBV DNA also could be detected in the medium and cell lysate of HUMSCs, but levels gradually decreased (Fig. 3b). If HUMSCs were passaged after incubation with human sera with HBV, the HBV DNA levels were significantly decreased in medium and cell lysate of HUMSCs (Fig. 3c).

## Discussion

In this study, we investigated the susceptibility of HUMSCs to HBV infection and detecting HBV in HUMSCs derived from HBV-infected donors using existing screening methods approved for clinic use. HBV antigens (HBsAg and HBeAg) and HBV DNA were assayed in the medium and cell lysate of HUMSCs derived from HBV-infected women using ELISA and FQ-PCR kits, respectively. Both HBV antigens (HBsAg and HBeAg) and HBV DNA failed to be detected in culture medium and cell lysate of HUMSCs derived from HBV-infected women. The ddPCR assay, the next-generation technology of quantitative PCR methods, was used to detect HBV copy number in the medium and cell lysate. HBV DNA failed to be detected in the culture medium and cell lysate of HUMSCs derived from HBV-infected women. Next, HUMSCs were incubated with human sera with HBV, and HBV DNA could be detected and had a stable level in the medium and cell lysate of HUMSCs at indicated time points without medium change and passage treatment. HBV DNA also could be detected in the medium and cell lysate of HUMSCs, but levels gradually decreased from day 0 to day 8, with medium change every 2 days. HBV DNA levels were significantly decreased, even with no detection in medium and cell lysate of HUMSCs after passage.

In stem cell therapy, the stem cell donors must be excluded for infectious diseases such as HBV, HCV, HIV I and II, syphilis, and CMV [17] and derived stem cells also must be screened for infectious pathogens [21]. In the present study, 14 maternal blood samples from donors were collected before delivery and were investigated for infectious diseases. Serological HBV markers including HBsAg, anti-HBs, anti-HBsAg, HBeAg, anti-HBe, and anti-HBc were assayed using an ELISA kit approved for clinical use. Serological examinations showed that two donors (No. 1 and No. 2) were HBV-infected patients and the others were healthy (No. 3–No. 14). HBV is highly species specific and classically considered to be a hepatotropic virus [22, 39]. Some studies have shown that HBV is not strictly hepatotropic and can be detected in extrahepatic tissues and cells such as PBMCs [23] and lymph nodes [25]. However, in contrast, several other studies have found that the HBV is not replicated in peripheral blood mononuclear cells (PBMCs) [40] or lymphatic tissues [41]. Therefore, the existence of extrahepatic replication of HBV is controversial. We wondered whether HBV could be detected in HUMSCs derived from HBV-infected women, and thus HUMSCs were isolated from 12 healthy and two HBV-infected women for further HBV detection in culture medium and cell lysate. However, we failed to detect HBV antigens (HBsAg and HBeAg) and HBV DNA in culture medium and cell lysate of HUMSCs derived from HBV-infected women using HBV screening kits approved for clinic use. HBsAg and HBeAg presence in serum shows different stages of HBV infection [42]. HBsAg, HBeAg, and HBcAb presence in serum at the same time suggests HBV replication. Cell infection with HBV is characterized by the presence of HBsAg [22, 42, 43]. HBeAg is usually present as an early serum marker of HBV infection and correlated with HBV DNA in serum [44]. The infant is more likely to be infected with HBV during the perinatal period if the mother is HBeAg-positive [22, 42]. Although the latest stem cell guidelines recommended that stem cells should undergo screening assay for infectious pathogens, our results showed a discrepancy with this recommendation in HUMSCs because we failed to detect HBV in HUMSCs derived from donors with HBV replication using FQ-PCR and even ddPCR.

MSCs were first discovered by Friedenstein et al. in the 1960s from BM, and MSCs obtained from BM are still regarded as the gold standard for clinical utilities [45]. However, it is reported that HBV could infect and replicate in human bone marrow mesenchymal stem cells (BMMSCs) [46]. We further investigated the susceptibility of HUMSCs to HBV infection. HUMSCs derived from healthy donors were incubated with human blood serum with a high titer of HBV. After 24 h of incubation, HBV DNA could be detected, with a stable level in medium and cell lysate of HUMSCs without medium change and passage treatment. However, HBV DNA levels gradually decreased with medium change every 2 days and were significantly decreased, even with no detection after passage. These results indicated that the positive detection of HBV DNA was mainly attributed to HBV attachment of HUMSCS and HBV could not replicate in HUMSCS in vitro. As shown in Fig. 2, the minimum threshold of the HBV FQ-PCR kit was 100 IU/ml, so HBV DNA assay results of these samples lower than 100 IU/ml were not valid and accurate. Thus, the current HBV DNA detection kit approved for clinical diagnosis was not applicable to HBV detection of isolated HUMSCs.

Currently, quality evaluation for stem cells is far from being established due to the limited understanding of stem cell sciences and the lack of relevant detection techniques [21]. Market-available HBV detection kits are mostly semiquantitative and designed for detection of blood samples. Development of applicable screening detection kits with a higher sensitivity for stem cell samples is needed. Thus, serological tests for infectious diseases from donors are more reliable than infectious pathogen detection from derived stem cells, at least for HBV. It seems to be unnecessary to detect HBV in derived HUMSCs using existing assay kits approved for clinical use because even when we used ddPCR, the current most sensitive method for DNA screening, we still failed to detect HBV DNA in HUMSCs derived from HBV patients.

## Conclusion

HBV failed to be detected in HUMSCs derived from HBV-infected donors and healthy HUMSCs incubated with HBV-positive human sera using an existing screening assay approved for clinical use. It is more crucial to screen out HBV using serological examination in maternal blood, comparing with a secondary cell-based HBV screening assay in HUMSCs derived for cell therapy. In addition, to solve the problem of occult hepatitis B infection (OBI) and window period (WP) infection, we recommend a more stringent donor eligibility determination in which the donor should undergo another serological test for infectious diseases 3 months later, before clinical use of the HUMSCs.

## Abbreviations

BMMSC: Bone marrow mesenchymal stem cell
ddPCR: Droplet digital PCR assay
ELISA: Enzyme-linked immunosorbent assay
FITC: Fluorescein isothiocyanate
FQ-PCR: Quantitative fluorescence PCR
GVHD: Graft versus-host disease
HBV: Hepatitis B virus
HCV: Hepatitis C virus
HIV: Human immunodeficiency virus
HUMSC: Human umbilical cord-derived mesenchymal stem cell
PBMC: Peripheral blood mononuclear cell
PE: Phycoerythrin
